# Kinase Inhibitors’ Effects on Innate Immunity in Solid Cancers

**DOI:** 10.3390/cancers13225695

**Published:** 2021-11-14

**Authors:** Chunying Peng, Katrin Rabold, Willem J. M. Mulder, Martin Jaeger, Romana T. Netea-Maier

**Affiliations:** 1 Department of Internal Medicine, Division of Endocrinology, Radboud University Medical Center, 6525 GA Nijmegen, The Netherlands; peng.chunying@radboudumc.nl (C.P.); katrin.rabold@radboudumc.nl (K.R.); 2 Radboud Institute for Molecular Life Sciences (RIMLS), Radboud University Nijmegen Medical Center, 6525 GA Nijmegen, The Netherlands; martin.jaeger@radboudumc.nl; 3 Department of Internal Medicine and Radboud Center for Infectious Diseases, Radboud University Medical Center, 6525 GA Nijmegen, The Netherlands; willem.mulder@radboudumc.nl; 4 Laboratory of Chemical Biology, Department of Biochemical Engineering, Eindhoven University of Technology, 5600 MB Eindhoven, The Netherlands

**Keywords:** kinase inhibitors, immune checkpoint inhibitors, VEGFR, immunogenic cell death, pyroptosis, tumor-associated macrophages, MDSCs, tumor microenvironment

## Abstract

**Simple Summary:**

In this review, we evaluate the updated data of the immunological effects of kinase inhibitors on the innate immune system and provide an in-depth analysis of the underlying mechanisms. We also discuss how this immunological effect can be harnessed to improve cancer treatment and highlight recent successes, such as the combination with anti-tumor immunotherapy. Last, we explore novel kinase targets and the incorporation of them with targeted drug delivery techniques as promising research areas.

**Abstract:**

Innate immune cells constitute a plastic and heterogeneous cell population of the tumor microenvironment. Because of their high tumor infiltration and close interaction with resident tumor cells, they are compelling targets for anti-cancer therapy through either ablation or functionally reprogramming. Kinase inhibitors (KIs) that target aberrant signaling pathways in tumor proliferation and angiogenesis have been shown to have additional immunological effects on myeloid cells that may contribute to a protective antitumor immune response. However, in patients with malignancies, these effects are poorly described, warranting meticulous research to identify KIs’ optimal immunomodulatory effect to support developing targeted and more effective immunotherapy. As many of these KIs are currently in clinical trials awaiting approval for the treatment of several types of solid cancer, we evaluate here the information on this drug class’s immunological effects and how such mechanisms can be harnessed to improve combined treatment regimens in cancer.

## 1. Introduction

The abnormal activation of signaling pathways is one of the hallmarks of various types of cancer. Kinases function as a crucial node within the signaling network by phosphorylating surface receptors and cytoplasmatic enzymes, activating cellular functions that could potentially trigger carcinogenesis. Therefore, protein kinases serve as a promising target for anti-cancer therapy. Following the success of the first small molecule kinase inhibitor (KI), Imatinib, for the treatment of chronic myelogenous leukemia 20 years ago, along with a better understanding of the molecular mechanism of oncogenesis, an increasing number of KIs have gone through extensive investigation, which has ultimately led to the broad clinical use of KIs in cancer patients with specific mutations.

Initially, the therapeutic efficacy of KIs has been postulated to be derived from their actions on malignant cells only. However, clinical evidence suggests that patients show durable responses after the discontinuation of KI therapy [1]. Supporting this notion, in preclinical studies, KIs exert stronger anti-tumor activity in immunocompetent animal models compared to immunodeficient mice, indicating that at least part of the anti-tumor activity is mediated via the immune system [2].

Immune evasion and suppression remain the major hurdles in anti-cancer therapy. Immunotherapy has revolutionized the treatment of cancer, giving hope to patients who were previously deemed incurable. However, only a minority of patients experience long-lasting benefits from these therapies, and the incidence of severe side effects remains high [3]. To unlock immunotherapy’s full potential, we must reinforce our therapeutic focus towards the immune system and its response to cancer. The current cancer immunotherapy strategies primarily focus on adaptive immune responses. For example, immune checkpoint blockades, such as cytotoxic T-lymphocyte-associated protein 4 (CTLA4) and programmed cell death protein 1/programmed cell death ligand 1 (PD1/PD-L1), activate T cells. However, the complex tumor microenvironment (TME) and associated myeloid cells’ immunosuppressive nature are responsible for the primary resistance to immune checkpoint inhibition (ICI) [4,5]. Since the onset and maintenance of T cell responses and the development of long-term protective memory depend on the interplay between the adaptive and innate immunity, targeting innate immunity provides a promising opportunity to re-activate the anti-tumor immune response [6]. Therefore, the role of the innate immune response in cancer immunotherapy should be considered in cancer immunology and immunotherapy.

The immunological effects of KIs provide a strong rationale for the combination with immune checkpoint inhibition, aiming at synergistic effects. In this review, we evaluate the most recent data on the immunological effects of KI, with a specific focus on the innate immune system. We will explore how these effects can be incorporated in the multimodal approaches against malignant tumors and to improve the patient outcome.

## 2. Innate Immunity in the Context of Cancer

**Figure 1:**
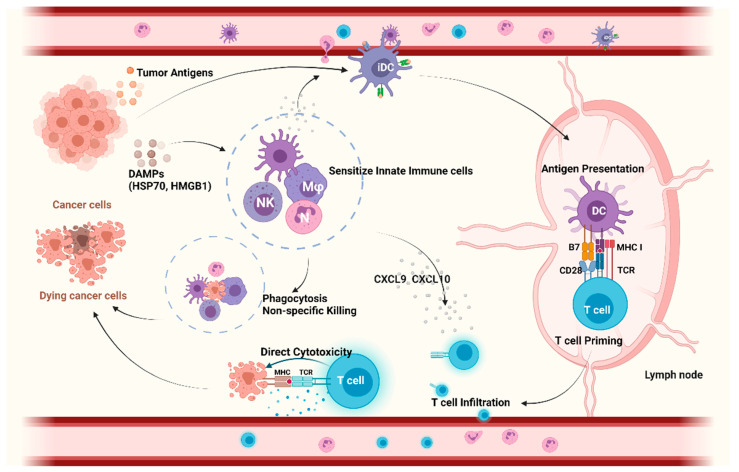
The role of innate immunity in anticancer response. Within the tumor immunity cycle, myeloid cells (NK cells, Mφ) recognize tumor cells via DAMPs and exert direct cytolysis by which they activate an adaptive immune response and recruit DCs into the TME. DCs take up and process tumor antigens and migrate to lymph nodes to cross-present the tumor antigens to CD8+ T cells, resulting in their activation. In addition, DCs produce chemokines to recruit tumor-specific CD8 T cells into TME, exerting direct cytotoxicity on tumor cells. Collectively, innate immune cells are closely involved in antitumor response via non-specific killing and modulation of tumor-specific T cell priming, infiltration, and cytotoxicity. Abbreviations: APC, antigen presenting cells; CXCL, Chemokine (C-X-C motif) ligand 9; DAMPs, damage-associated molecular pattern; DCs, dendritic cells; HMGB1, high mobility group box 1 protein; HSP70, 70 kilodalton heat shock proteins; iDC, immature dendritic cells; Mφ, macrophage; MHC, major histocompatibility complex; NK, natural killer cells; TCR, T cell receptor; TME, tumor microenvironment. Created with BioRender.com.

In addition to providing protection against infections, the innate immune system serves as the front line of the non-specific defense against oncogenesis and cancer progression, but it also initiates and directs the adaptive immune response (Figure 1). Cells of the innate immune system, such as natural killer (NK) cells, rapidly detect membranous or soluble molecules from tumor cells by pattern recognition receptors (PRRs) and exert direct cytolysis on tumor cells. Next to their direct cytotoxic effects, they orchestrate downstream inflammatory reactions. Ultimately, this leads to the infiltration of other myeloid and lymphoid cells, such as dendritic cells (DCs), into TME. Macrophages and DCs function as professional antigen-presenting cells (APCs). After tumor antigen uptake, DCs migrate to secondary lymphoid organs to cross-present the tumor antigens and prime tumor-specific CD8+ T cells, resulting in their activation [7]. In addition, DCs in the tumor produce chemokines that are essential for the infiltration of tumor-specific CD8+ T cells into the tumor that exert direct tumor cytotoxicity [8].

Tumor-associated macrophages (TAMs) are the largest fraction of the myeloid infiltration within TME, which is generally associated with adverse clinical outcomes, as shown in various solid tumors [9,10,11,12,13]. Under the influence of different local factors, such as cytokines and chemokines, macrophages recruited in the TME undergo functional programming, resulting in an immunosuppressive and pro-tumoral phenotype [14]. The underlying spectrum of TAMs’ immunosuppressive properties has not been completely elucidated and ranges from direct effects on tumor cell proliferation and angiogenesis expansion to the recruitment of T regulatory cells (Treg) and suppression of cytotoxic CD8+ T cells [15]. Re-educating TAMs towards an antitumor phenotype might unleash the cytotoxic function of CD8+ T cells; hence, clinical trials examining the combination of macrophage antagonists and other immunotherapies, such as ICIs, are ongoing (e.g., NCT04301778) [16,17]. Furthermore, the inhibition of macrophage differentiation and recruitment by blocking macrophage colony-stimulating factor signaling improves anti-CTLA4 and anti-PD1 therapeutic efficacy [16]. In addition to TAMs, populations of myeloid cells with immunosuppressive properties have also been described in the circulation and termed ‘myeloid-derived suppressor cells’ (MDSCs). They are recruited to the TME by several chemokines, such as C-C motif ligand (CCL) 2 and CCL5, where they rapidly differentiate into TAMs in various preclinical tumor models [18]. Similar to TAMs, the MDSC recruitment inhibition improves the response to ICIs in two different murine carcinoma models [19]. Interestingly, in murine tumor models, it was shown that anti-PD-1 monoclonal antibody (mAb) therapy rapidly removed from PD-1+ CD8+ T cells and transferred to neighboring PD-1 negative TAMs, which could be reversed by the blockage of the Fc/FcγR binding that eventually amplifies anti-PD-1 therapeutic efficacy [20]. In cancer, different types of innate immune cells, including TAMs and MDSCs, facilitate tumor growth and counteract anti-tumoral adaptive immune responses. Regulating innate immune responses, therefore, offers compelling opportunities for long-lasting, comprehensive tumor control.

## 3. Kinase Inhibitors (KIs) for Cancer Treatment

**Figure 2:**
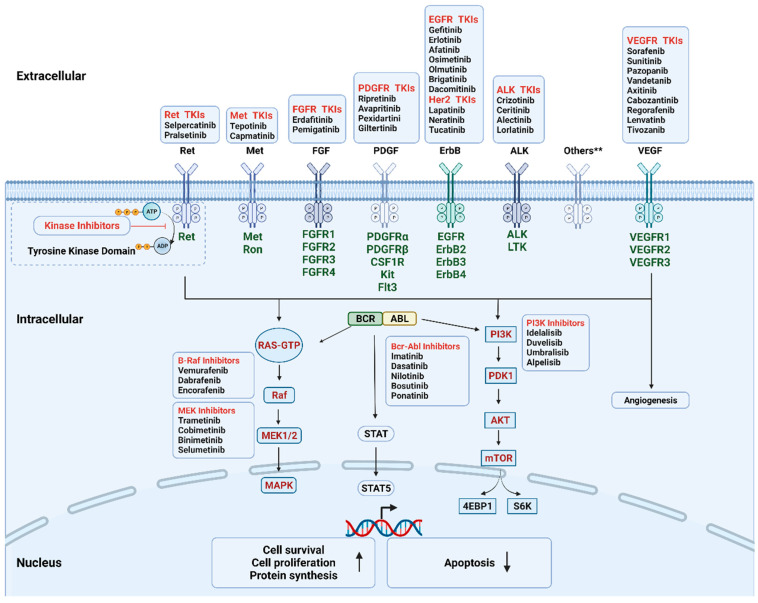
Mechanism of the FDA-approved kinase inhibitors. The targeted kinases include receptor tyrosine kinase (cell surface, in dark green), non-receptor tyrosine kinase (intracellular, BCR-ABL), and serine/threonine protein kinase (intracellular, in red). During oncogenesis and angiogenesis, constitutive overexpression or activation of protein kinases promotes cell proliferation and survival and inhibits apoptosis. Kinase inhibitors can pass through cell membrane and interact with the intracellular domain by interfering with the transfer of the terminal phosphate of ATP to the substrates, hence blocking the activation of the downstream signaling cascades. Others** include AXL family, Trk family. Abbreviations: Akt, protein kinase B; EGFR, epidermal growth factor receptor; FGFR, fibroblast growth factor receptors; MAPK, mitogen-activated protein kinase; mTOR, mechanistic target of rapamycin; PDGFR, platelet-derived growth factor receptor; PDK1, phosphoinositide-dependent kinase-1; PI3K, phosphoinositide 3-kinase.; STAT, signal transducer and activator of transcription; TKIs, tyrosine kinase inhibitors; VEGFR, vascular endothelial growth factor receptor. Created with BioRender.com.

During the past few decades, molecular pathways regulated by driver oncogenes have been extensively studied. We now know that genetic alterations of protein kinases are involved in the oncogenesis of various types of cancer. Therefore, oncogenic kinases have become highly attractive pharmaceutical targets over the past two decades [21]. The targeted kinase group includes tyrosine kinase and serine/threonine protein kinase; both contain surface receptors and cytoplasmic signaling proteins (Figure 2). Receptor tyrosine kinases (RTKs) serve as the receptor of multiple growth factors and cytokines, while downstream cytoplasmic signaling kinases, such as MEK1/2, transmit signals to the nucleus [22,23]. KIs are developed as a group of small molecules that can pass through the cell membrane and interact with the intracellular ATP binding site of kinases, leading to the blockage of various downstream signaling cascades. Until now, the U.S. Food and Drug Administration (FDA) has approved more than fifty KIs for clinical use in cancer patients in the United States. The majority of KIs target RTKs, while others target intracellular signaling kinase and serine/threonine protein kinases (Table 1). The most common drug targets include vascular endothelial growth factor receptor (VEGFR), epidermal growth factor receptor (EGFR), BCR-ABL, and anaplastic lymphoma kinase (ALK) [22].

The advantage of targeted therapies, such as KIs, compared with conventional chemotherapy, is that they are highly specific, which may limit treatment-related side effects. However, as the targets of KIs are widely distributed, the receptor cross-reactivity between the targeted cancer cells and healthy cells in patients could cause off-target toxicities, such as cytopenia and gastrointestinal side effects [24].

Based on target selectivity profiles, the development of KIs has followed two related yet independent paths [25,26,27], namely multitargeted kinase inhibitors (MKI) and selective inhibitors. The overall anti-tumor activity of MKIs originates from the concomitant inhibition of a broad spectrum of human kinome. The clinical indication of these drugs does not necessarily require additional individualized patient selection. A notable example is Sorafenib, which has been approved for the treatment of advanced renal cell carcinoma (RCC), hepatocellular carcinoma (HCC), and thyroid cancer due to its broad inhibitory profile, including B-Raf, VEGFRs and fibroblast growth factor receptors (FGFR), platelet-derived growth factor receptors (PDGFR), etc. [28]. On the other hand, the highly selective KIs tend to inhibit a specific component of the oncogenic pathway. Patients are often selected for treatment with these drugs based on specific predictive biomarkers using clinical samples. For example, the EGFR tyrosine kinase inhibitor (TKI) Gefitinib is recommended as the first-line treatment of non-small cell lung cancer (NSCLC) patients with EGFR sensitizing mutations [29].

KIs were initially developed as tumor-intrinsic therapies; however, recent work uncovered that they are capable of modulating the composition and functionality of the tumor immune microenvironment [30]. Preclinical and clinical data indicate that KIs, at clinically relevant concentrations, exert immunomodulatory effects (Table 2): they can synergize with immunotherapy or impair host immune defense, leading to secondary infections. Thus, they could have either a favorable or detrimental impact on clinical outcomes [24,31,32,33]. However, early phase drug development has mainly employed human cancer cell lines that were either cultured in vitro or xenografted into immunodeficient hosts; therefore, the potential immunological effect was overlooked. It is reasonable to assume that only those with favorable immunological effects have been selected for further clinical trials. Thus, the impact of a targeted therapy-induced stress response on tumor cells and the corresponding anti-tumor immunity remains to be further investigated. 

## 4. Immunological Actions of Kinase Inhibitors through Effects on Tumor Cells

The immunological effect of KIs could derive from the direct impact of drugs on malignant cells. As a notable example, KIs may invoke immunogenic cell death (ICD), an immunologically unique type of apoptosis that could elicit a potent adaptive immune response against dead cell-related antigens. Unlike the homeostatic removal of dying cancer cells (i.e., tolerogenic cell death), which facilitates an anti-inflammatory and tumor-promoting process, ICD induced by certain anticancer agents may elicit the immunostimulatory clearance of dying cells by APCs, where the dying cancer cells function as a vaccine that drives tumor antigen-directed immunity [55]. The capacity of inducing ICD has been observed in chemotherapy with agents, such as doxorubicin and oxaliplatin, previously [56]. More recently, it has been reported that Crizotinib, a multitargeted VEGFR TKI, was able to induce ICD in a NSCLC model [34]. Interestingly, compared to Crizotinib single-agent treatment, a combination of Crizotinib and cisplatin, a non-ICD inducing chemotherapeutical agent, further sensitized NSCLC to immunotherapy by upregulating PD-1/PD-L1 in a murine lung adenocarcinoma model [55]. Similar results have been obtained in a murine prostate cancer model. Cabozantinib, a combinatorial VEGFR and c-MET inhibitor, exerted a greater anti-tumor effect than c-MET inhibition alone, suggesting a potential mechanism independent of c-MET inhibition. It has been found that Cabozantinib triggers the release of C-X-C motif chemokine ligand 12 (CXCL12) and high mobility group box 1 protein (HMGB1) from dying tumor cells, which mediate acute neutrophil mobilization and migration, contributing to an amplified anti-tumor response. Although the ICD-induced anti-tumor response ultimately relies on CD8+ T cells, intact toll-like receptor 4 (TLR4) signaling in tumor-infiltrating innate immune cells, such as DCs, is required since the danger signals or TLR agonists are first perceived by innate immune cells. Thus, TLR4 functions as a crucial node that bridges innate and adaptive immunity in the process of ICD [57].

Another mechanism of the immunogenic effects of KIs is through the induction of pyroptosis, a hyperinflammatory type of programmed cell death that was initially observed in macrophages upon infection [58]. The immunomodulatory potential of pyroptosis is mediated through the secretion of pro-inflammatory cytokines, such as interleukin (IL)-1β and endogenous DAMPs, during the process of cell rupture. Nevertheless, Erkes et al. revealed that melanoma cells treated with a BRAF–MEK inhibitor released factors that could activate and prime the DCs in a pyroptosis-dependent way [38]. Indeed, in an immunocompetent murine model, the BRAF–MEK inhibitor treatment increased the expression of multiple immunostimulatory molecules, including HMGB1, calreticulin, and IL-1α, from melanoma cells, which led to increased major histocompatibility complex (MHC) -II expression on the tumor-resident DCs [38].

KIs could also interfere with the paracrine signaling between tumor and immune cells as cytokines released by cancer cells can have diverse effects on myeloid cell function. For instance, in a prostate cancer murine model, tumor cells were able to recruit MDSCs and drive the immunosuppression-related gene expression in these cells through the secretion of multiple cytokines. This effect was abrogated by Cabozantinib pre-treatment by downregulating the immunosuppressive cytokine levels, including IL-1 receptor antagonist (IL-1Ra), CCL 5, CCL12, CD40, and hepatocyte growth factor (HGF) [40].

Altogether, these observations support the idea that the anti-tumor immune response elicited by tumor-released factors/signals upon KI treatment might have profound clinical implications.

## 5. Immunological Effects of VEGFR–MKIs

VEGFR is the major therapeutic target for most MKIs. The multitarget VEGFR KIs can exert an immunological effect either through the alteration of angiogenesis in the TME or through other non-angiogenic effects on the TME.

### 5.1. Immunologic Effects of VEGFR–MKIs Related to Angiogenic Alterations of the TME

A structurally abnormal and leaky vasculature is a hallmark of most tumor tissues. VEGF, released by tumor cells and surrounding stoma, is the major driver of tumor angiogenesis. The VEGFR family contains three tyrosine kinases, of which VEFGR2 is the dominant signaling receptor for VEGFs that transmits signals to the nucleus and ultimately promotes vascular endothelial cell mitogenesis, survival, and vascular permeability during angiogenesis [59]. VEGFRs are expressed by various cell types within the TME, mostly endothelial cells but also immune cells [60,61]. Beyond their role in angiogenesis, the VEGF/VEGFR pathways are also implicated in modulating both local and systemic immunosuppression. The underlying mechanism could be mediated, directly or indirectly, by vasculature. First, VEGF has been shown to inhibit DC maturation and antigen presentation, thus hampering T cell activation [62]. Second, the overexpression of VEGF creates an abnormal vascular network in cancer, contributing to an immunosuppressive TME marked by hypoxia, low pH, and elevated interstitial pressure, which could facilitate the recruitment and differentiation of the immunosuppressive cells, including TAMs, MDSCs, and Tregs. It has been shown that MDSCs would rapidly differentiate into immunosuppressive TAMs in hypoxic conditions [18,63]. Therefore, the immunostimulatory effect of anti-VEGF therapy could derive from normalizing tumor vasculature, alleviating hypoxia, increasing effector T cell infiltration, decreasing the population of MDSCs and Tregs, and restoring DCs [64,65,66]. Supporting this notion, various preclinical studies revealed that anti-VEGF therapy, either with anti-VEGF mAb or TKIs, could enhance the efficacy of different immunotherapies, leading to tumor regression and a prolonged animal survival phenotype [67,68]. Based on those promising results, such combinations have been widely investigated in clinical trials [69].

### 5.2. Immunological Effects of VEGFR–MKIs Independent of the Angiogenic Pathway

While many of the immunological effects of VEGFR–MKIs can be explained by their VEGFR-inhibitory effects, various other cellular and environmental factors within TME may be involved as well. It has been well established that KIs have a direct impact on both malignant and other healthy cells. The tumor infiltrating myeloid cells (TIMCs) are of particular interest because some of the targeted kinases are also expressed on TIMCs [70]. Indeed, some VEGFR–MKIs have shown multiple immunostimulatory actions via the direct interaction with myeloid cells or altering the TME. Such effects include promoting the functional programming of TAMs towards an antitumoral phenotype [46,64], restoring the activity of circulating monocytes [45], suppressing the population of regulatory T cells [71], and inhibiting the function of MDSCs [47,48].

Supporting the therapeutic relevance of these mechanisms, accumulating evidence suggests that MKIs promote an immune permissive environment by the direct immune-stimulation on myeloid cells. For instance, Sorafenib activated NK cell-mediated direct killing against a tumor by inducing pyroptosis in the macrophages and reducing the MHC-I expression on malignant cells simultaneously [49]. Along similar lines, a murine HCC model showed that Sorafenib activates NK cells via the reprogramming of TAMs towards an anti-tumor phenotype [46].

Besides triggering the non-specific cytolysis function of NK cells, KIs mediate an immune-stimulatory effect by priming effector T cells. For instance, in a preclinical murine model, low-dose Regorafenib was found to increase cytotoxic T cell function and antitumor immunity through the programming of macrophages toward the anti-tumor phenotype, possibly via the p38 mitogen-activated protein kinase (p38MAPK) pathway [39]. DCs play a central role in the initiation and sustaining of the anti-tumor T cell response. Upon taking up dying tumor cells that release DAMPs, DCs undergo maturation, migration, and then process cancer antigens onto MHC-I for presentation to CD8+ T cells. Tumors have developed multiple pathways to suppress DC maturation and function, including the release of VEGF. Sorafenib restores DC maturation partially by the blockage of the VEGF pathway [50]. Interestingly, similar results have been obtained using Pazopanib, which promoted DC differentiation and functionality by upregulating the maturation markers MHC-II, CD40, and C-C chemokine receptor type 7 (CCR7) while downregulating PD-L1 concomitantly [51].

Neutrophils are the first line responders in the initiation of an innate immune response, and can acquire either pro-inflammatory, antitumor, or pro-tumorigenic properties through mobilization and infiltration. The differentiation of the aforementioned phenotypes requires the close interaction with the chemokine context of TME, which could potentially be modulated by MKIs [72]. For instance, Cabozantinib triggers a neutrophil-mediated anticancer activity via CXCL-12/HMGB1/CXCR4-mediated recruitment in a murine model [52]. Similarly, Sorafenib treatment recruits tumor neutrophil infiltration in animal models as well as HCC patients by inducing the CXCL5 expression in tumor cells via the hypoxia-inducible factor 1-alpha (HIF1α)/nuclear factor-κB (NF-κB) pathway [53].

MDSCs are described as a heterogeneous population of pathologically activated myeloid cells at various stages of maturation. MDSCs affect multiple aspects in TME, including immune suppression, promotion of angiogenesis, and tumor invasion; thus, the functional alterations caused by KIs might assist or impair their efficacy [73]. Supporting this notion, an in vitro study showed that Nilotinib, Dasatinib, and Sorafenib impaired the MDSCs formation in the early induction phase [47]. On the contrary, in a murine orthotopic HCC model, Sorafenib treatment increased MDSCs infiltration [48]. Moreover, contradictory findings have also been observed in clinical studies. Data from HCC patients revealed that the PD-L1 expression in tumor infiltrating macrophages was significantly increased after Sorafenib treatment. These conflicting observations might be caused by the difference between the murine and human immune system and difficulties in replicating human TME, thus, further investigation is warranted [44,48].

In summary, the majority of the FDA-approved MKIs have been shown to mediate the robust immune-stimulatory effects that might amplify the therapeutic anti-tumor efficacy. However, inconsistencies in the literature should be noted, possibly because of the difference in the selectivity and targeted kinome among the MKIs and the relative shortage of clinical studies. Hence, the therapeutic relevance remains a matter of debate.

## 6. Immunomodulation by Other Kinase Inhibitors

Besides the VEGFR pathway, many other growth factors and RTKs have been shown to work in a complementary and coordinated manner to regulate tumor growth and angiogenesis. Although practically all kinase inhibitors have been reported to exert antitumor activity somehow via an immune-mediated mechanism [74], certain kinase families are of particular interest since they are involved in both oncogenic and immune-related pathways.

### 6.1. AXL Pathway

The TYRO3, AXL, and MERTK (TAM) family is a group of RTKs that uniquely co-express on immune cells. Besides its role in tumorigenesis and metastasis, the TYRO3/AXL/MERTK family plays a major role in maintaining tissue homeostasis and wound healing via various mechanisms, including efferocytosis, reprogramming macrophages towards anti-inflammatory phenotypes, and terminating TLR signaling in APCs. The TYRO3/AXL/MERTK family can be triggered by apoptotic cell materials, which are abundant within the TME, hence promoting a immunosuppressive landscape [75]. Therefore, targeting the TYRO3/AXL/MERTK RTK family may directly impact tumor growth and relieve immunosuppression [76]. Supporting this notion, the high expression of AXL is associated with a resistance to ICIs in a murine breast cancer model [77]. In line with these observations, several preclinical studies have shown the antitumor immunomodulatory effects of Cabozantinib, a small molecule KI targeting the AXL and MET pathways. The possible mechanism includes increased MHC-I expression and robust neutrophil infiltration and the concomitant suppression of MDSCs recruitment [78].

### 6.2. HGF/c–MET Axis

Functioning as an RTK binding with HGF, c-MET is aberrantly expressed in cancers, regulating cell proliferation, motility, migration, and invasion [79]. Interestingly, pre-clinical data have demonstrated the potential immunomodulatory effect of c-MET inhibitors. Particularly, Capmatinib, a c-MET kinase inhibitor approved by the FDA to treat metastatic NSCLC with specific mutations, has shown to promote an anti-tumor response independent of the tumor cell-intrinsic c-MET pathway blockade. Further, c-MET is also expressed by tumor stromal cells. For instance, neutrophils upregulates c-MET expression in tumor-bearing conditions [80]. In a murine melanoma model, c-MET+ neutrophils acquired an immunosuppressive phenotype after being recruited into the TME. The adjuvant inhibition of the c-MET pathway either by Capmatinib or genetic ablation specifically in neutrophils resulted in the improved efficacy of immunotherapy and restored T cell expansion and functionality [43]. Nonetheless, contradictory results have been reported in both animal models and patient samples, showing an increased expression of the MET on neutrophils compared to healthy controls, suggesting that the therapeutic benefit of the MET inhibition in cancer cells could be, to some extent, hindered by the concomitant blockade of the c-MET pathway in anti-tumor neutrophils [54].These findings underscore a mixed role of MET KIs in cancer treatment: on the one hand, in tumors with MET proto-oncogene mutation, this pathway is vital for the cell survival; on the other hand, it promotes tumor-associated neutrophil (TAN) infiltration. However, due to the limited information available on the roles of TAN, the MET inhibitor mediated immunological response remains to be further elucidated.

### 6.3. The Downstream MAPK Pathway and mTOR Pathway

Activated RTKs are able to recruit and regulate a wide range of intracellular protein kinases to regulate cell proliferation and differentiation, including RAS/MAPK and PI-3K/AKT/mTOR signaling. Those cytoplasmic kinases are crucial mediators of extracellular signals perceived by RTKs, and oncogenic mutations in these kinases are also common in various solid tumors [81]. The MAPK pathway is an essential oncogenic driver of human malignancies and targeted therapy; specifically, blocking this signaling module has widely been employed as an important anti-tumor strategy [82]. Trametinib is an inhibitor targeting MEK1/2, two related kinases in the MAPS cascade. In an immunocompetent murine model, Trametinib restored an immune-permissive environment by blocking the expansion of monocyte-MDSCs. This is the result of a reduced differentiation of MDSCs from their bone marrow precursors and a decreased secretion of chemotactic molecules from tumor cells that contribute to the reduced recruitment of the MDSC in the TME [83]. Along similar lines, Selumetinib, an ERK inhibitor, has been reported to relieve immunosuppression in a murine model. Selumetinib impaired the recruitment and differentiation of tumor-promoting infiltrating immune cells, including TANs, granular MDSC, and Ly6C + MHCII+ intermediate TAMs within TME, while concomitantly inhibiting the expression of environmental mediators of immunosuppression, such as cyclooxygenase (Cox)-2 and arginase (Arg)-1 [41].

Taken together, these observations complemented and extended the findings of the immunomodulation of KIs, which could serve as possible therapeutic targets or be used as combinatorial regimes with other immunotherapies.

## 7. Rationale of the Combined Therapy

**Figure 3:**
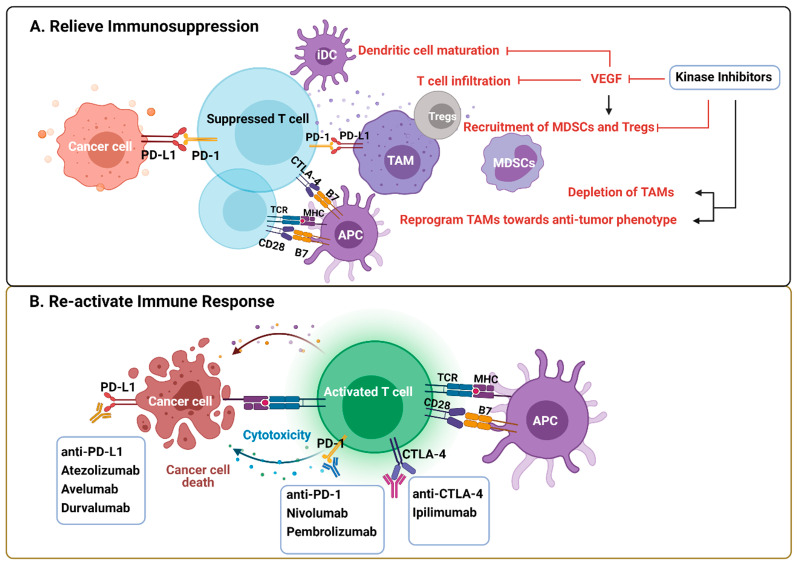
Rationale of kinase inhibitors and ICI combined therapies. (**A**) VEGF produced by tumor cells and immune cells creates a tumor-promoting and immunosuppressive environment by impeding DC maturation and T cell infiltration, increasing the recruitment of Tregs, TAMs, and MDSCs. KIs exert immunological effects by either blocking VEFG pathway or depleting/reprogramming the immunosuppressive cells directly. (**B**) ICI are antibodies against inhibitory pathways that block effective antitumor T cell response, including the PD-1/PD-L1 axis and CTLA-4. The rationale of the combined therapy is predicated on relief of immunosuppression within TME by KIs and re-activating T cell response by ICI. Abbreviations: CTLA-4, cytotoxic T-lymphocyte-associated protein 4; ICI, immune checkpoint inhibitors; KIs, kinase inhibitors; MDSCs, myeloid derived suppressor cells; PD-1, programmed cell death protein 1; PD-L1, programmed death-ligand 1; TAM, tumor-associated macrophages; Tregs, regulatory T cells. Created with BioRender.com.

Immunotherapies, especially ICI that targets PD-1/PD-L1 and CTLA-4 pathways to re-activate T effector cells, play an emerging role in cancer treatment [84]. However, trials done in advanced melanoma, NSCLC, and RCC indicate that only a small fraction of the patients benefit from these treatments [85,86,87]. Therefore, combination therapies are needed to increase the response rate. Unresponsive patients tend to have non-T-cell-related intratumoral inflammation, characterized by the tumor-permissive and immunosuppressive phenotypes of innate immune cells [88]. The immunological effects mediated by KIs potentially influence their clinical performance, on the one hand by re-activating the immune response that enhances their efficacy or by inducing immunosuppression that offsets the efficacy of KI monotherapy but may be therapeutically actionable by ICI (e.g., Sorafenib induced PD-L1 upregulation) [44] (Figure 3). Thus, the combination of ICI and targeted anti-cancer agents, such as KIs, are promising immune-oncology multimodal strategies.

Preclinical studies have shown the synergistic antitumor efficacy of such combinations rely on the innate immune system [2,40,89]. For example, Cabozantinib was shown to enhance ICI efficacy through selectively depleting MDSCs via the suppression of MDSC-promoting cytokines released by cancer cells and the upregulation of IL-1Ra [40]. Similarly, Regorafenib amplified the antitumor efficacy of ICI through both anti-angiogenic and VEGFR-independent mechanisms [39,90]. On the other hand, the addition of ICI could overcome the undesirable immunological effects caused by KI. This is exemplified by the combination of Lenvatinib-anti-PD-1/PD-L1 in a pre-clinical immunocompetent murine model, in which Lenvatinib monotherapy increased the infiltration of TAMs, Tregs, and polymorphonuclear MDSCs (PMN-MDSCs), while combined therapy showed a significantly decreased infiltration by PMN-MDSCs, which, in turn, was associated with an improvement in tumor shrinkage and survival [35]. Besides ICI, other immunotherapy, such as a cancer vaccine, could also benefit from the immunological effects of KIs when administered in combination. For instance, Sorafenib augmented the antitumor effects of mouse chimeric antigen receptor (CAR) T cells in an immunocompetent murine model, in part by promoting IL-12 secretion in TAMs and cancer cell apoptosis [36]. Similarly, Sunitinib showed a synergic effect with cancer vaccination by depleting MDSCs, which reversed de novo resistance to ICI [42]. In an in vitro and subcutaneous animal model, Foretinib upregulated the PD-L1 expression in tumor cells through the JAK2-STAT1 pathway while compromising the function of diverse immunosuppressive cells, such as TAMs and MDSCs, simultaneously, hence synergizing with the anti-PD-1 antibody to enhance the T cell anti-tumor response through relieving the immunosuppressive factors within the TME [37].

Several trials using combined ICI and KI therapies are ongoing or have been completed (summarized in Table 3). Despite the promising results from the preclinical studies, the regimen of Sunitinib or Pazopanib combined with an anti-PD-1 antibody was largely offset by its excessive side effects (e.g., liver toxicity). Therefore, the combination with a more selective VEGFR–MKI, like Axitinib, has been investigated [91,92]. Although most of the clinical trials are still in the early phases, several international multi-centered randomized controlled trials (RCTs) have been conducted based on the tolerable dose and safety profile obtained from phase I/II trials. The development of a consensus and guidelines is currently underway. RCC is a highly VEGF-dependent malignancy that facilitates the use of VEGF–MKIs as the standard therapy for advanced patients; however, the resistance to single agent therapy inevitably develops in almost all patients [93]. Even though the mechanism of resistance remains unclear, the early phase trials of MKI–ICI combined therapy showed a higher response rate and prolonged survival [94], leading to the large-scale investigation of MKI–ICI combination in advanced patients. Specifically, the CLEAR trial has demonstrated an improved outcome of Lenvatinib–Pembrolizumab over Sunitinib [95] with regard to the objective response rate and progression of free survival. Similar results have been obtained in another two independent trials investigating Cabozantinib plus Nivolumab and Axitinib plus Pembrolizumab [96,97]. As a result, the latest RCC guidelines recommend three MKI–ICI combinations for the treatment of advanced RCC [98].

## 8. Future Prospects/Remaining Questions

As detailed above, the broad immunomodulatory properties of KIs, combined with the impressive progress in understanding cancer pathophysiology and the development of new immunotherapeutic approaches, sets us up for improving cancer patient treatments and outcomes. Several questions remain, while the answers to these questions through future research in the coming years will open new opportunities in cancer treatment.

The first area of future research in this field is to understand even better, and at a deeper level, the biological effects of KIs. Understanding the precise molecular pathways through which these drugs influence immune function lies at the foundation of identifying new therapeutic targets for more potent KIs, or even new compounds targeting equivalent molecules. Another important area of investigation must focus on KIs’ most important immune cell targets. Subsequently, due to the prominent role of the TME in tumor progression and immune evasion, significant opportunities exist for the development of novel KIs in restoring an immune-permissive TME. A notable example is tyrosine–protein kinase MER (MERTK), a member of the TYRO3/AXL/MERTK RTKs family, one of the latest identified oncogenes with concomitant immunoregulatory functions in the innate immune system, due to its broad expression in various TIMCs responsible for wound healing and maintaining homeostasis under physiological conditions [75,76,99,100]. Previous studies demonstrated that the immunosuppressive effects of MERTK consist of multiple aspects, including the induction of an anti-inflammatory cytokine profile (e.g., transforming growth factor beta (TGF-β), IL-10, HGF), modulation of the PD-1/PD-L1 axis, and functional reprogramming of macrophages, MDSCs, NK cells, and T cells. Preclinical studies have shown promising results of MERTK inhibitors. For instance, MRX-2843, a selective MERTK-inhibitor, has proven effective in MERTK-negative acute lymphoblastic leukemia (ALL) and NSCLC in immunocompetent models, suggesting immune-mediated therapeutic activity, which was granted an early phase of clinical trials in patients (e.g., NCT04872478, NCT04762199) [101]. More recently, the other two candidates of the MERTK inhibitor, ONO-7475 and INCB081776, are also in early stages of clinical trials (e.g., NCT03176277, NCT03522142). Of note, colony stimulating factor 1 receptor (CSF1R), a class III RTK, plays a crucial role in shaping macrophage plasticity and phenotypic heterogeneity. The blockade of CSF1R signaling could be a promising anti-cancer therapy, presumably by depleting the TME of immunosuppressive macrophages and releasing anti-tumor immune responses [102] However, most of the drug development is still in the early stage, with limited success in clinical cancer trials. To our knowledge, at present, Pexidartinib is the only FDA-approved CSF-1R small molecule kinase inhibitor for the treatment of a rare benign disease-tenosynovial giant cell tumor, in 2019 [103,104], while broader clinical utility warrants further investigation.

Secondly, another important area of investigation for the clinical oncologists is to try and understand the heterogeneity of the therapeutic response to KI treatment. We should investigate the factors influencing this heterogeneity (why do some patients respond better than others?) and how we can identify these patients (what are the biomarkers that can tell us beforehand that a patient responds effectively to KI therapy?).

Thirdly, despite these successes, the field still requires a better understanding of how to fully exploit KIs for a therapeutic benefit. On the one hand, the poor accumulation at tumor sites and severe off-target effects that cause intolerable systemic side effects in patients have greatly offset their therapeutic efficiency and limited further clinical application. Nanoparticles that improve the selective delivery of drug payloads to the cancer cells or tumor-promoting immune cells would offer a great opportunity to overcome the drug toxicity profile and amplify antitumor activity, as recently shown in experimental models [105]. On the other hand, we should investigate and screen which combinations of KI-containing regimens are most effective for which type of cancer: it is conceivable to hypothesize that, for some types of tumors, combinations between KIs and other forms of immunotherapy would be effective (e.g., ICIs), while, for other types of cancer, KI treatment should be combined with chemo- or radiotherapy.

In conclusion, in the present review, we highlighted the myeloid cells’ indispensable role in anti-tumor immune responses, and the effect KIs exhibit to modulate these important immunological mechanisms. We described KIs’ potential to improve combination therapies for solid cancers, and we anticipate that ongoing clinical trials will ultimately guide the implementation of optimal combinatorial approaches to maximize the immune system’s anti-tumor activity.

## Figures and Tables

**Table 1:** List of FDA-approved kinase inhibitors for solid cancers.

Targets	Clinical Indications	Kinase Inhibitors
**Receptor Tyrosine Kinase**		
VEGFR (multi-targeted)	RCC, HCC, GIST, DTC, MTC, CRC	Sorafenib (2005), Sunitinib (2006), Pazopanib (2009), Vandetanib (2011), Axitinib (2011), Cabozantinib (2012), Regorafenib (2012), Lenvatinb (2015), Tivozanib (2021)
EGFR	Lung cancer	Gefitinib (2002), Erlotinib (2004), Afatinib (2013), Osimetinib (2015), Olmutinib (2016), Brigatinib (2017), Dacomitinib (2018)
ALK	Lung cancer	Crizotinib (2011), Ceritinib (2014), Alectinib (2014), Lorlatinib (2018)
MET	Lung cancer	Tepotinib (2019), Capmatinib (2020)
RET	Lung, thyroid cancer	Selpercatinib (2020), Pralsetinib (2020)
Her2	Breast Cancer	Lapatinib (2007), Neratinib (2017), Tucatinib (2020)
Kit, PDGFR, CSF1R, FLT3	GIST	Ripretinib (2020), Avapritinib (2020)
	TGCT	Pexidartinib (2019)
FGFR	Bladder, cholangiocarcinoma	Erdafitinib (2019), Pemigatinib (2020)
Trk	Any metastatic solid tumor with NTRK mutations	Larotrectinib (2018), Entrectinib (2019)
**Serine/threonine Protein Kinase**		
CDK family	Breast cancer	Palbociclib (2015), Ribociclib (2017)
B-Raf, MEK1/2 pathway	Melanoma	Vemurafenib (2011), Dabrafenib (2013), Trametinib (2013), Cobimetinib (2015), Encorafenib (2018), Binimetinib (2018)
	Neurofibromatosis type I	Selumetinib (2020)
PI3K pathway	Breast Cancer	Alpelisib (2019)

FDA-approved small molecule kinase inhibitors for the treatment of solid cancer up to 2021; kinase inhibitors are categorized based on primary targets and clinical indications. Abbreviations: ALK, Anaplastic lymphoma kinase; CRC, Colorectal cancer; CSF1R, Colony stimulating factor 1 receptor; DTC, Differentiated thyroid cancers; EGFR, epidermal growth factor receptor; FGFR, fibroblast growth factor receptors; FLT3, Fms like tyrosine kinase 3; GIST, Gastrointestinal stromal tumors; Her2, Human epidermal growth factor receptor 2; MTC, Medullary thyroid cancer; NSCLC, Non-small-cell lung carcinoma; PDGFR, Platelet-derived growth factor receptors; PI3K, Phosphoinositide 3-kinases; Raf, Rapidly accelerated fibrosarcoma; RCC, Renal cell carcinoma; RTKs, Receptor tyrosine kinases; SLL, Small lymphocytic lymphoma; TGCT, Tenosynovial giant cell tumor; TKIs, Tyrosine kinase inhibitors.

**Table 2:** Immunological effects of kinase inhibitors in preclinical models.

Impact on Immune Cells	Drug	Tumor Type	Model	Impact on Tumor Cells	Combination with Immunotherapies	Efficacy of Combination with Immunotherapy	Ref.
Increases proinflammatory Mφ and T cell infiltration, Upregulates PD-1 and CTLA-4 expression	Crizotinib 10µM (plus cisplatin)	NSCLC	Transplantable tumor in immunocompetent murine model	Induces ICD, Upregulates PD-L1 expression	Anti-PD-1 Anti-CTLA-4	Reaches 100% cure rate	[34]
Decreases tumor-infiltrating monocytes and macrophages. Increases CD8+ T cells	Lenvatinib	HCC	Immunocompetent murine model (compared to immunocompromised)		Anti-PD-1	Improves tumor regression	[2]
Increases tumor-infiltrating macrophages, CD8+ T-cells, Tregs, PMN-MDSCs	Lenvatinib	ATC	Orthotopic tumor murine model		Anti-PD-1	Improves tumor reduction	[35]
Increases IL12 secretion from TAMs	Sorafenib	HCC	Immunocompetent murine model (compared to immunocompromised)	Induces cancer cell apoptosis	GPC3-targeted chimeric antigen receptor T cell therapy	Increases animal survival	[36]
Decreases TAMs, Promotes T cells infiltration	Foretinib	Colorectal Carcinoma	Subcutaneous tumor in murine model	Increases PD-L1 expression on tumor cells	Anti-PD-1	Improves tumor regression, prolongs overall survival	[37]
Reduces TAMs and MDSCs in TME Promotes T cell expansion	BRAF inhibitors + MEK inhibitors	Melanoma	Immunocompetent murine model (compared with immunocompromised model)	Induces pyroptosis	ND		[38]
Reprograms Mφ towards anti-tumor phenotype	Regorafenib	HCC	In vitro		Anti-PD1	Reduces tumor growth, Improves animal survival	[39]
Selectively depletes MDSCs	Cabozantinib, BEZ235	Prostate cancer	Spontaneous tumor in immunocompetent murine model	Inhibits PI3K pathway; Reduces CCL5, CCL12, CD40, HGF, Increases IL-1ra, CD142, and VEGF released by tumor cells	Anti-CTLA-4 + Anti-PD-1	Decreases tumor size, Reduces lymph node metastasis and lung micro-metastasis	[40]
Decreases infiltration of granulocytic MDSCs and neutrophils	Selumetinib	Colorectal	Transplantable tumor in murine model		Anti-CTLA-4	Reduces tumor volume, Prolongs animal survival	[41]
Depletes MDSCs Increases CD8^+^ T cells	Sunitinib	HPV-Induced cancer	Induced tumor in murine model		Cancer vaccine	Increases survival rate	[42]
Impairs recruitment of immunosuppressive TAN, enhances T cell expansion	Capmatinib	Melanoma Lung Breast Colon	Transplantable tumor + Inducible primary tumor in murine model		Anti-PD-1, PCP	Increases animal survival	[43]
Increases PD-L1 expression in TAMs	Sorafenib	HCC	Patient tissue		ND		[44]
Upregulates IL-15Ra expression on circulating monocytes	Sorafenib	Melanoma	Patient tissue		ND		[45]
Favors the expansion of proinflammatory TAMs, activates antitumor response of NKs	Sorafenib	HCC	Murine HCC model + In vitro		ND		[46]
Negatively affects the differentiation of monocytes into functional MDSCs	Sorafenib	NA	In vitro		ND		[47]
Increases MDSCs infiltration	Sorafenib	HCC	Orthotopic liver or subcutaneous tumor in murine model		ND		[48]
Induces Mφ pyroptosis, activates NK cells anti-tumor response	Sorafenib	HCC	Spontaneous and transplantable murine model + in vitro		ND		[49]
Restores DCs maturation	Sorafenib	NA	In vitro		ND		[50]
Improves DCs differentiation and performance	Pazopanib	NA	In vitro		ND		[51]
Increases neutrophil infiltration and anti-tumor activity	Cabozantinib	Prostate cancer	Genetic engineered tumor murine model	Triggers release of CXCL12 and HMGB1 from dying tumor cells	ND		[52]
Increases TANs infiltration	Sorafenib	HCC	Transplantable murine model + patient tissue biopsy		ND		[53]
Reduces anti-tumor TANs recruitment	MET TKIs (PF-04217903, INCB28060, JNJ-38877605)	Melanoma	Transplantable tumor in Met conditional knockout mice		ND		[54]

Notes: studies were included if they evaluated a kinase inhibitor in preclinical setting in solid cancers and reported effects on myeloid cells (TAMs, monocytes, MDSCs, NK cells, DCs, and neutrophils); studies were excluded if the drug was designed for the treatment of hematological malignancies. Abbreviations: ATC, anaplastic thyroid cancer; CTLA-4, cytotoxic T-lymphocyte-associated protein 4; CCL5, chemokine ligand 5; CCL12, chemokine ligand 12; CXCL-12, chemokine ligand 12; DC, dendritic cells; HCC, hepatocellular carcinoma;HMGB1, high mobility group box 1; HPV, human papillomavirus; ICD, immunogenic cell death; Mφ, macrophages; NA, not applicable; ND, not determined; NK, natural killer cells; PCP, poly(I:C) + CpG; PD-1, programmed cell death protein 1; PD-L1, programmed death-ligand 1; PI3K, phosphoinositide 3-kinases; PMN-MDSCs, polymorphonucler myeloid-derived suppressor cells; TAN, tumor-associated neutrophils.

**Table 3:** Ongoing/completed phase 3 clinical trials of kinase inhibitors combined with immunotherapies in treatment of solid cancer.

Immunotherapy	Kinase Inhibitor Treatment	Conditions	Enrollment (Estimated)	NCT Number	Outcome Measures
Pembrolizumab (PD-1)	Lenvatinib	Endometrial Neoplasms	827	NCT03517449	OS, PFS
		HCC	750	NCT03713593	OS, PFS
		Malignant Melanoma	660	NCT03820986	OS, PFS
		Nonsquamous NSCLC	726	NCT03829319	OS, PFS
		NSCLC	620	NCT03829332	OS, PFS
		Endometrial Neoplasms	875	NCT03884101	OS, PFS
		Urothelial Carcinoma	694	NCT03898180	OS, PFS
		Metastatic NSCLC	405	NCT03976375	OS, PFS
		HNSCC	500	NCT04199104	OS, PFS, ORR
		HCC	950	NCT04246177	OS, PFS
		Advanced/Metastatic GEA	790	NCT04662710	OS, PFS
		RCC	1431	NCT04736706	OS, PFS
		CRC	434	NCT04776148	OS
		Advanced/Metastatic RCC	1069	NCT02811861 *	Prolonged PFS (23.9 vs. 9.2 m) Improves OS ** (HR, 0.66)
Pembrolizumab	Axitinib	Advanced/Metastatic RCC	861	NCT02853331 *	Prolonged PFS (15.1 vs. 11.1 m) Improves OS (89.9% vs. 78.3%) Increases ORR (59.3% vs. 35.7%)
Pembrolizumab	Encorafenib	Melanoma	624	NCT04657991	PFS
Atezolizumab (PD-L1)	Cabozantinib	HCC	740	NCT03755791	OS, PFS
		RCC	500	NCT04338269	OS, PFS
		Metastatic Prostate Cancer	580	NCT04446117	OS, PFS
		NSCLC	350	NCT04471428	OS
Atezolizumab	Sorafenib/Lenvatinib	Unresectable HCC	554	NCT04770896	OS
Nivolumab (PD-1)/Ipilimumab (CTLA-4)	Cabozantinib	Metastatic RCC	1046	NCT03793166	OS
		RCC	840	NCT03937219	PFS
Nivolumab (PD-1)	Cabozantinib	Advanced/Metastatic RCC	638	NCT03141177 *	Prolonged PFS (16.6 vs. 8.3 m) Increases OS (85.7% vs. 75.6%) Increases ORR (55.7% vs. 27.1%)
Nivolumab (PD-1)	Sitravatinib	Metastatic Non-Squamous NSCLC	532	NCT03906071	OS
Avelumab (PD-L1)	Axitinib	RCC	888	NCT02684006	OS, PFS
IMA901 (cancer vaccine)	Sunitinib	Metastatic RCC	339	NCT01265901	OS

*: Completed trials. Results indicate the outcomes of combination therapy (KI+ immunotherapy) compared to standard KI monotherapy OS **: 12 months follow-up for the measurement of overall survival. Abbreviations: GEA, gastroesophageal adenocarcinoma; HNSCC, head and neck squamous cell carcinoma; HR, hazard ratio for disease progression or death; m, months; ORR, objective response rate; OS, overall survival; PFS, progression-free survival.
